# Exploring Topics, Emotions, and Sentiments in Health Organization Posts and Public Responses on Instagram: Content Analysis

**DOI:** 10.2196/70576

**Published:** 2025-05-02

**Authors:** Abigail Paradise Vit, Avi Magid

**Affiliations:** 1 Department of Information Systems The Max Stern Emek Yezreel College Jezreel Valley Regional Council Israel; 2 Management Rambam Healthcare Campus Haifa Israel; 3 Department of International Health Maastricht University Maastricht The Netherlands

**Keywords:** emotion analysis, fear, health communication, health care, Instagram, official health organizations, sentiment analysis, social media, vaccines

## Abstract

**Background:**

Social media is a vital tool for health organizations, enabling them to share evidence-based information, educate the public, correct misinformation, and support a more informed and healthier society.

**Objective:**

This study aimed to categorize health organizations’ content on social media into topics; examine public engagement, sentiment, and emotional responses to these topics; and identify gaps in fear between health organizations’ messages and the public response.

**Methods:**

Real data were collected from the official Instagram accounts of health organizations worldwide. The BERTopic algorithm for topic modeling was used to categorize health organizations’ posts into distinct topics. For each identified topic, we analyzed the engagement metrics (number of comments and likes) of posts categorized under the same topic, calculating the average engagement received. We examined the sentiment and emotional content of both posts and responses within the same topic, providing insights into the distributions of sentiment and emotions for each topic. Special attention was given to identifying emotions, such as fear, expressed in the posts and responses. In addition, a linguistic analysis and an analysis of sentiments and emotions over time were conducted.

**Results:**

A total of 6082 posts and 82,982 comments were collected from the official Instagram accounts of 8 health organizations. The study revealed that topics related to COVID-19, vaccines, and humanitarian crises (such as the Ukraine conflict and the war in Gaza) generated the highest engagement. Our sentiment analysis of the responses to health organizations’ posts showed that topics related to vaccines and monkeypox generated the highest percentage of negative responses. Fear was the dominant emotion expressed in the posts’ text, while the public’s responses showed more varied emotions, with anger notably high in discussions around vaccines. Gaps were observed between the level of fear conveyed in posts published by health organizations and in the fear conveyed in the public’s responses to such posts, especially regarding mask wearing during COVID-19 and the influenza vaccine.

**Conclusions:**

This study underscores the importance of transparent communication that considers the emotional and sentiment-driven responses of the public on social media, particularly regarding vaccines. Understanding the psychological and social dynamics associated with public interaction with health information online can help health organizations achieve public health goals, fostering trust, countering misinformation, and promoting informed health behavior.

## Introduction

### Background

Social networks are used by billions of people around the world, making them an effective platform for reaching a wide range of audiences. Health organizations, such as the World Health Organization (WHO), use social networks to disseminate important health-related information [1-5]; provide real-time updates, news, and emergency guidelines [6]; and promote awareness of diseases [7] and mental health [8]. Health organizations also promote vaccination compliance by disseminating information regarding vaccination importance, safety, and disease severity [9].

In contrast to the content published by social network users unaffiliated with health organizations, who can spread a large amount of inaccurate, false, and misleading information about health-related issues [10,11], the health-related content published on social networks by official health organizations enables the public to have access to reliable and useful information [12-14]. Therefore, health organizations are important actors in social media as reliable sources, providing evidence-based and authoritative information [15]. Through their efforts, these organizations educate the public, dispel myths, and help the community become healthier and more informed [16].

In addition to serving as a means of disseminating health-related information, social networks provide the public with the opportunity to participate in discussions and conversations with health organizations [17] by posting comments, sharing posts, and liking posts. Members of the public can also ask questions about the health issue being discussed, and the organizations that disseminate the information can respond. Active public participation can enhance individuals’ understanding of health-related content [18,19] and can foster public trust in and appreciation for science [20]. In addition, health organizations are able to pinpoint concerns related to specific health topics, gain insight into public opinion [17-25], and identify topics that result in misinformation [26].

However, health-related messages disseminated by health organizations can provoke negative public reactions [27]. When combined with contradictory messages from unaffiliated social media users, this can lead to undesired health behaviors, such as vaccine hesitancy, noncompliance with health directives, and diminished trust in the reliability of health organizations [28-30].

Understanding public emotions in response to information disseminated by health organizations on social media is important for assessing the effectiveness of health communication strategies [31]. Sentiment and emotion analysis are widely used for determining sentiment polarity and detecting specific emotions expressed in textual data [32]. Sentiment analysis categorizes a text as positive, negative, or neutral, while emotion analysis identifies specific emotions expressed within a text [32]. Fundamental emotions, such as happiness, sadness, anger, disgust, surprise, and fear, can be detected, along with more nuanced emotions such as confusion and trust [33,34]. Among these emotions, fear is an important factor in health communication, as it can influence public perception, engagement, and behavioral responses [35].

Various theoretical models, such as the extended parallel process model [36], have explored how fear is used in public health messaging to encourage protective behaviors. This model suggests that individuals respond to fear-based messages depending on their perceived threat level and their sense of efficacy in managing the threat. According to this model, when perceived risk is high and the message also offers a clear solution, individuals are more likely to engage positively and adopt protective behaviors. However, if either condition is not met, responses may be negative, leading to outcomes such as avoidance or denial [36]. Therefore, fear is a crucial factor to consider in public health communication.

Despite the need to examine how the public emotionally responds to information shared by health organizations on social media, studies examining the topics communicated by these organizations and the corresponding responses from the public are sparse. The aim of this study is to analyze and characterize the content disseminated by health organizations on social media into topics, as well as social media users’ responses to this content and the engagement, sentiment, and emotions induced by this content. In addition, we aim to identify gaps in fear between health organizations’ messages and public responses.

### Related Work

In this section, we provide an overview of various studies associated with health-related content and the topic modeling and sentiment analysis of such content.

#### Sentiment Analysis of Health-Related Social Media Content

Many studies examined the sentiment and public opinion surrounding the COVID-19 vaccine on Twitter [21,23,24,37-53]. For example, an analysis of Twitter data was conducted by Niu et al [24] to examine public opinion and sentiment before and during the administration of the COVID-19 vaccines in Japan. They found that negative sentiment toward the vaccines dominated positive sentiment in Japan, and concerns about side effects may have outweighed fears of infection at the beginning of the vaccination process.

Numerous studies leveraged machine learning techniques to classify tweets as positive, negative, or neutral sentiment toward vaccines, enabling the identification of vaccine hesitancy among communities and social media users [35,54-68]. Most of these studies collected data from Twitter using keywords or hashtags related to vaccinations. Chakraborty et al [56] used deep learning to analyze 226,668 COVID-19 tweets from December 2019 to May 2020, achieving 81% accuracy. Most tweets showed positive or neutral sentiment, while highly retweeted posts were predominantly neutral or negative.

#### Topic Modeling for Health-Related Social Media Content

Several studies have used topic modeling to examine health-related discussions on social media, focusing on topics such as blood donation [69], cancer-related content [70], and vaccine-related conversations [71]. Paul and Dredze [72] proposed the ailment topic aspect model to identify health topics on Twitter. Analyzing 144 million tweets, they identified 13 topics linked to seasonal influenza, allergies, temporal surveillance, and obesity-related geographic data in the United States.

Seltzer et al [73] analyzed 500 Instagram (Meta Platforms, Inc) images tagged #zika from May to August 2016, analyzing them by sentiment, content, and engagement. A total of 299 images were related to health, while 193 focused on topics of public interest. Sentiments and emotion analysis revealed that fear and negative emotions were linked to Zika transmission and response uncertainty. The study highlighted Instagram’s value in understanding public sentiment and addressing gaps in health communication. Furthermore, Muralidhara and Paul [74] analyzed 96,426 Instagram posts collected between September and October 2016, using 269 health-related hashtags. Polylingual topic modeling approach was used to identify 47 health-related topics spanning 10 broad categories: acute illness, alternative medicine, chronic illness and pain, diet, exercise, health care and medicine, mental health, musculoskeletal health and dermatology, sleep, and substance abuse. Kim et al [75] analyzed 96,302 Instagram photos and 513,694 comments with antivaccination hashtags, focusing on photo features, engagement, and sentiment. Most photos (52.24%) were categorized as “text.” “Food” and “plant” photos received the most positive comments, while “text” photos, despite high engagement, received fewer positive responses.

Other studies focused on dividing vaccine content on social networks into topics [40,41,43,45,52,53,76]. The study by Kwok et al [76] examined tweets of Australian users regarding COVID-19 vaccination on Twitter. Using a latent Dirichlet allocation topic model, they identified 3 commonly discussed topics: attitudes toward COVID-19 and vaccination, advocacy for infection control measures against COVID-19, and misconceptions and complaints regarding COVID-19. Similarly, Lyu et al [40] used latent Dirichlet allocation to analyze COVID-19 vaccine discussions on Twitter, identifying 16 topics grouped into 5 themes. Vaccination opinions were the most discussed topic. Emotion analysis showed trust as the dominant emotion, followed by anticipation, fear, and sadness. In addition, Chandrasekaran et al [43] used the correlation explanation topic modeling algorithm to examine COVID-19 vaccine–related tweets. The authors identified 16 topics in the COVID-19 vaccination tweets, which were grouped into 6 broader themes. Most tweets regarding COVID-19 vaccination centered on vaccine policy, vaccine hesitancy, and postvaccination symptoms and side effects.

#### Analysis of Health Care Providers’ Content and Public Responses on Social Media

Several studies have examined content published on social networks by health care providers. Among them, Kim and Kim [77] analyzed 1545 Instagram photos published by the US Centers for Disease Control and Prevention (CDC) and public comments using Microsoft Azure Cognitive Services. Their findings showed that most images featured text or people, but those with larger faces or flashy elements tended to receive less engagement. Happiness and neutral emotions in comments were negatively correlated with interaction levels. Pinto et al [78] analyzed 632 Instagram posts from Portugal’s National Health Service (NHS) and Brazil’s Ministry of Health (MH) in 2019, mapping 53 topics for the NHS and 63 for the MH. The NHS emphasized healthy eating and blood donation, while the MH focused on vaccination campaigns, dengue prevention, and HIV awareness.

Mello et al [79] analyzed 726 Instagram posts from the WHO and CDC in 2020 to explore how these organizations communicated COVID-19 risks. Their study focused on messaging related to threat and efficacy, cues to action, and indicators of credibility. According to the findings, efficacy messages, such as those promoting preventive behaviors, were more prevalent, while threat messages addressing the susceptibility and severity of COVID-19 were less common. The study concluded that improving credibility cues, using compelling visuals, tailoring content for diverse audiences, and leveraging Instagram’s interactive features could enhance public health communication, boosting engagement, trust, and impact.

Vaghefi et al [80] analyzed health care providers’ messages on Twitter from May 2018 to May 2019 using machine learning, including Bidirectional Encoder Representations from Transformers (BERT)–based models, to classify tweets as professional communications or health-related information, further categorizing them as fear based or hope based. Results showed that fear-based messages were widely shared by the public but were less effective at motivating constructive health actions, while hope-based messages resonated more with health care providers. While this study [80] examined health care providers’ content and the fear evoked by their messages on Twitter, our study focuses on categorizing health care providers’ posts into distinct topics; analyzing engagement metrics, sentiment, and emotional content within each topic; and identifying the gaps between the fear expressed in messages from health organizations and the fear observed in public responses to such content. In contrast, Vaghefi et al [80] did not analyze the public comments to measure fear in relation to health care providers’ messages; instead, they focused on public interactions, such as retweets and replies, to study information propagation.

## Methods

### Overview

**Figure 1:**
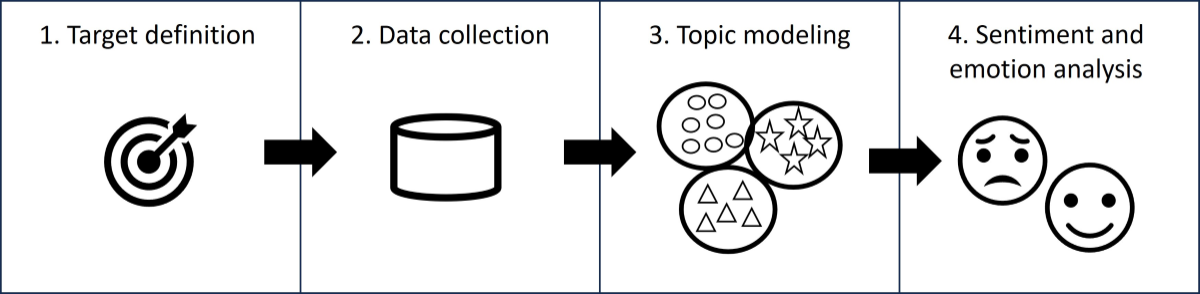
An overview of the methodology’s main phases.

This section outlines the proposed methodology, which consists of four main phases, as illustrated in Figure 1: (1) defining the targeted health organizations, (2) gathering data from the selected health organization accounts, (3) performing topic modeling on health organization content, and (4) analyzing the sentiment and emotion associated with each topic and the public’s response to it.

Detailed descriptions of each phase are provided in the subsections that follow.

### Target Definition

The first phase of our methodology was to define the group of health organizations to be examined. These organizations were selected based on their status as official government entities or government-supported health agencies responsible for public health policies, research, and regulations, ensuring that the study focused on authoritative sources [81-88]. In addition, a minimum threshold of 10,000 followers was set for health organization accounts to ensure sufficient activity and public engagement.

A brief description of each organization is provided in Textbox 1.

**Description of the health organizations included in this study.**

**Description**

1. The World Health Organization (WHO): the WHO is the United Nations agency tasked with connecting nations around the world to promote health, keep the world safe, and serve populations considered vulnerable [81]. The organization’s official Instagram account is named WHO.
2. Department of Health and Human Services (HHS): the HHS aims to enhance the health and well-being of the residents or citizens of the United States by providing effective health and human services and fostering sound, sustained advances in the sciences underlying medicine, public health, and social services [82]. HHSgov is the official Instagram account of the HHS.
3. The Office of Minority Health (OMH): the OMH is part of the HHS dedicated to improving the health of racial and ethnic minority groups. The OMH fulfills its commitment to improving the health of racial and ethnic minority groups in large part by developing health policies and programs that help eliminate health disparities [83]. MINORITYHEALTH is the official Instagram account of the OMH.
4. National Institutes of Health (NIH): the NIH is the leading federal agency in the United States responsible for conducting and supporting medical research. The NIH, which is part of the HHS, is one of the world’s most prominent centers for medical research. Its mission is to enhance human health by advancing research across a wide range of scientific disciplines [84]. The organization’s official Instagram account is named NIHgov.
5. The National Institute of Mental Health (NIMH): the NIMH is the US agency responsible for research on mental health. Its primary objective is to understand, treat, and prevent mental illness by conducting basic and clinical research. The NIMH is one of the 27 institutes and centers that comprise the NIH, which is part of the HHS [85]. NIMHgov is the official Instagram account of the NIMH.
6. The US Centers for Disease Control and Prevention (CDC): the CDC is a science-based, data-driven organization that leads the United States’ efforts to protect the public’s health. The CDC is one of the major components of the HHS, and it aims to protect the residents of the United States from health, safety, and security threats, both foreign and domestic [86]. CDCgov is the official Instagram account of the CDC.
7. The UK National Health Service (NHS): the NHS was established as the public health care system of the United Kingdom. It is one of the largest and most comprehensive health care systems in the world. The NHS provides various types of services, including mental health services, general practitioners, hospitals, and treatment facilities. The NHS is dedicated to improving the overall health of the population by promoting public health initiatives, health education, vaccination programs, and disease prevention campaigns [87]. The organization’s official Instagram account is named NHS.
8. The US Food and Drug Administration (FDA): the FDA is a governmental regulatory agency responsible for protecting public health by ensuring the safety, efficacy, and security of human and veterinary drugs, biological products, and medical devices [88]. The organization’s official Instagram account is named FDA.

It is important to note that, because we selected health organizations with a high number of followers, the resulting sample skewed toward US-based organizations. Most accounts originated from the United States, with 1 from the United Kingdom and 1 global organization (WHO). This concentration may affect the generalizability of the findings, as both the published content and the public responses are likely influenced by US-specific health priorities and cultural context.

### Data Collection

We chose Instagram as the social media platform for its visual and interactive nature, which allows health organizations to share information and engage with the public effectively [89]. We searched for the official Instagram accounts of health care organizations using Instagram’s search box. Table 1 provides the name of each official Instagram account for the organizations, along with the total number of posts published since the account’s creation and the number of followers, as recorded in August 2024. As can be seen in Table 1, the CDC, WHO, and Office of Minority Health are the organizations that publish the most posts. The WHO’s account has the largest number of followers.

To collect the health organizations’ Instagram posts, we connected with the Instagram application programming interface (API) using the RapidAPI website. This website is a large API hub that allows to connect with tens of thousands of public Representational State Transfer APIs over the internet.

The posts were collected between April 7, 2017, and November 17, 2023. Each Instagram post included the publication date, the number of likes, the number of comments, text, and a photo. For each post, we collected the comments, including the publication date, the comment text, and the number of likes. A total of 6082 posts and 82,982 comments were collected using the Instagram API. All retrieved posts and comments were included in the analysis. Table 2 presents the relevant statistics for the collected posts and comments for each health organization’s Instagram account. We analyzed all the posts that the API allowed us to retrieve, without applying selection criteria or filtering specific posts. Comments were collected only from the original posts, excluding replies to comments.

**Table 1:** Statistics for the health organizations’ Instagram accounts, ranked by number of followers (highest to lowest).

Organization name	Instagram account name	Published posts, n	Followers, n
Centers for Disease Control	CDCGOV	6359	2.5 million
World Health Organization	WHO	3893	12 million
National Health Service	NHS	695	564,000
Department of Health and Human Services	HHSGOV	3269	202,000
Food and Drug Administration	FDA	801	122,000
National Institute of Mental Health	NIMHGOV	674	64,100
National Institutes of Health	NIHGOV	1867	279,000
Office of Minority Health	MINORITYHEALTH	3909	15,400

**Table 2:** Statistics for the collected posts and comments of each health organization’s Instagram account.

Name of health organization	Collected posts, n	Collected comments, n	Number of likes, mean (SD)
Centers for Disease Control	4298	58,471	2725.341 (3870.086)
World Health Organization	527	15,448	18349.774 (28197.828)
National Health Service	277	3826	1443.018 (1386.701)
Department of Health and Human Services	116	896	207.836 (270.549)
Food and Drug Administration	85	565	203.259 (183.758)
National Institute of Mental Health	301	1096	185.920 (142.871)
National Institutes of Health	253	1987	723.447 (709.083)
Office of Minority Health	225	603	34.124 (31.448)

### Ethical Considerations

The data collection process and analysis were approved by the Emek Yezreel College Ethical Review Board (2023-81 YVC EMEK).

As the research relied solely on publicly available social media data and did not involve direct interaction with individuals, informed consent was not applicable. No compensation was offered or provided, as the study did not involve direct participation of human participants.

No identifiable private user information was collected or analyzed. All data used in the analysis were publicly available and did not contain personally identifiable information.

### Topic Modeling

Text from the posts published by health organization accounts was analyzed using BERTopic [90]. Note that we removed the names of the health organizations and their abbreviations from the text to avoid creating topics around each organization. Stop words were also removed from the text.

We used the C_V_ coherence score to evaluate the quality and interpretability of the topics produced by the model. This measure combines the indirect cosine measure with normalized pointwise mutual information and a Boolean sliding window [91]. The coherence score indicates how closely related and coherent the words are in a topic. Using the average coherence metric, which is the average of the coherence metrics within each topic, we measured the model’s ability to generate coherent and meaningful topics. This score enabled us to evaluate the overall effectiveness of the model in producing topics with a high degree of semantic similarity.

We performed 6 steps in BERTopic to analyze the posts (Textbox 2).

**Six-step BERTopic analysis of posts.**

1. Embedding tweets: in this step, the text in the posts was converted into numerical representations using a sentence-transformers model named All-MiniLM-L6-v2 [92].
2. Dimensionality reduction: uniform manifold approximation and projection [93] was used to reduce the dimensionality of the embedded text, with the following parameters: _neighbors=15, n_components=3, min_dist=0, and metric = ‘cosine.’
3. Cluster tweets: the text was grouped into clusters using the hierarchical density-based spatial clustering of applications with noise density-based clustering technique [94], with the following parameters: min_cluster_size=40, metric=“Euclidean,” and cluster_selection_method=‘eom.’
4. Word frequency analysis in clusters: the frequency of each word in each cluster was determined at the cluster level.
5. Topic representation: to represent the topics in the Instagram posts and responses, term frequency–inverse document frequency (TF-IDF) was adapted to work on a cluster or topic level instead of a tweet level. A new TF-IDF representation was used called class-based TF-IDF (c-TF-IDF).
6. Outlier reduction: hierarchical density-based spatial clustering of applications with noise identified texts that were outliers, meaning they did not belong to any of the established topics. To address this, we calculated the c-TF-IDF representation for each outlier text and compared its cosine similarity with the c-TF-IDF representations of the existing topics. By associating outlier texts with the closest matching topic based on similarity, we minimized the number of texts classified as outliers.

### Sentiment and Emotion Analysis

Having categorized the health organizations’ posts into topics, we analyzed the emotions and sentiment of each post and comment associated with a particular topic. Sentiment analysis was conducted using *distilbert - base - multilingual - cased - sentiments - student*, which achieved an average accuracy of 0.808 on the test set [95]. This is a distilled version of a zero-shot classification pipeline trained on the multilingual sentiment dataset. Zero-shot classification is a machine learning technique that allows models to classify data into categories they have never encountered during training without requiring labeled examples to be provided. It accomplishes this by leveraging contextual understanding and semantic relationships between seen and unseen classes, often using embeddings or natural language models [96]. In this case, a larger “teacher” model, *MoritzLaurer/mDeBERTa-v3-base-mnli-xnli*, was used to train a smaller “student” model, *distilbert-base-multilingual-cased*. Using this distillation process, the student model maintains high classification performance while being more efficient and lightweight. According to the training log, the student model achieved an impressive agreement rate of 88.29% with its teacher model.

Emotion analysis of 6 basic emotions (fear, anger, disgust, joy, sadness, and surprise) was conducted using a fine-tuned checkpoint of the DistilRoBERTa-base model called *j-hartmann/emotion-english-distilroberta-base*. The model was trained on 6 diverse datasets [97]. The model was trained on a balanced subset from several datasets of nearly 20,000 observations in total. In total, 80% of this balanced subset was used for training and 20% for evaluation. The evaluation accuracy was 66%.

To ensure the accuracy of the models in identifying sentiments and emotions, we randomly selected 100 posts and comments. We manually classified them based on their dominant sentiment and dominant emotion. The results showed that the sentiment model achieved 92% accuracy, while the emotion model demonstrated 84% accuracy. Considering sentiment models predict binary classification and emotion models face greater complexity due to emotions’ multidimensional nature, the results are logical. Therefore, applying the models to the data was expected to provide sufficiently reliable outcomes.

Each post and posts’ comments received a sentiment score of positive, negative, or neutral, as well as an emotion score of fear, anger, disgust, joy, sadness, or surprise.

For each topic, we calculated the following:

- Average number of comments (the average number of comments for all posts in the topic).
- Average number of likes (the average number of likes for all posts in the topic).
- Average post sentiment scores (the average positive, negative, and neutral sentiment scores for all posts in the topic).
- Average post emotion scores (the average fear, anger, disgust, joy, sadness, and surprise scores for all posts in the topic).
- Average comment sentiment scores (the average positive, negative, and neutral sentiment scores for all post comments in the topic).
- Average comment emotion scores (average fear, anger, disgust, joy, sadness, and surprise scores were calculated for all post comments in the topic).
- Gap (the difference between the average fear score of the posts and the average fear score of the comments).

### Linguistic Analysis

The objective of this analysis was to identify the most significant phrases used by health organizations in posts that resonated more positively with the public (ie, associated with higher positive sentiment in comments) compared to those that elicited more negative reactions (higher negative sentiment in comments).

For this purpose, we calculated the average sentiment score (positive and negative) of all comments related to each post. Posts were then categorized based on the dominant sentiment (positive or negative) derived from these average sentiment scores in comments, resulting in 2 groups: posts with predominantly positive comments and posts with predominantly negative comments.

For each group of posts, we extracted the top 50 most significant phrases, including single words (unigrams), 2-word phrases (bigrams), 3-word phrases (trigrams), and 4-word phrases (four-grams). The preprocessing involved removing hashtags from the text, eliminating stopwords, and calculating term importance using TF-IDF. TF-IDF estimates the significance of terms within a group of posts based on their frequency within each post as compared to the frequency across all posts in the group. The cumulative TF-IDF scores for each term were calculated by summing across all posts in the group, enabling the identification of the most significant phrases for each group of posts.

### Time Analysis

In the time analysis section, we examined the average positive and negative sentiment in both comments and posts over the years. In addition, we analyzed the average levels of various emotions, including fear and anger, expressed in both posts and comments throughout the data collection period. Furthermore, we decided to calculate additional emotions of trust, disappointment, and confusion. The purpose of including these emotions was to extend the analysis beyond the 6 basic emotions, providing a broader range of emotional insights.

To calculate trust, we used the *ayoubkirouane/BERT-Emotions-Classifier* model [98], a fine-tuned BERT-based model designed for multilabel emotion classification. This model was trained on the sem_eval_2018_task_1 dataset. This model includes a wide range of emotions, such as anger, anticipation, disgust, fear, joy, love, optimism, pessimism, sadness, surprise, and trust.

For confusion and disappointment emotions, we used the *SamLowe/roberta-base-go_emotions* model [99], which is trained on the go_emotions dataset for multilabel classification. The go_emotions dataset, based on Reddit (Reddit, Inc) data, contains 28 emotion labels. The model achieved high performance in identifying confusion (with an accuracy of 0.972) and disappointment (with an accuracy of 0.974).

As part of the temporal analysis, we incorporated weekly COVID-19 mortality data in the United States using a publicly available dataset published by the National Center for Health Statistics [100]. The analysis included calculating a “COVID-19 death ratio,” which represents the proportion of US COVID-19–related deaths to the total number of US deaths. We applied a rolling average smoothing technique across a 10-week window to reduce noise and variability in the data, allowing us to identify patterns over time. This approach allowed us to combine the mortality data alongside our sentiment and emotional analyses, providing valuable insights into the alignment between public responses and real-world outcomes.

## Results

### Topic Modeling

Using BERTopic, 36 topics were identified for the posts of the 8 health organization Instagram accounts. The average coherence score was 0.7374. A health expert reviewed the list of topics and suggested that we combine related topics.

Therefore, the following topics were combined: 7 topics related to COVID-19 were grouped together, 2 topics related to vaccines for children were grouped together, 2 topics related to monkeypox were grouped together, and 2 topics related to booster vaccines were combined. Combining the topics resulted in 25 topics, with an average coherence score of 0.7298. The health expert assigned a representative name to each topic. The topic names were assigned by analyzing the 10 most significant words for each topic, as determined by the c-TF-IDF scores (refer to the Methods section for more information about c-TF-IDF) from the BERTopic algorithm and cross-referencing them with example posts related to that topic. Each topic was automatically assigned a unique number starting from 0, following the default numbering convention used by the BERTopic algorithm. Table 3 lists the topics, indicating their number, name, and number of posts. Refer to Multimedia Appendix 1 for the 5 words with the highest c-TF-IDF for each topic.

**Table 3:** The number, name, and size of each topic.

Topic number	Topic name	Topic size, n
0	COVID-19	1446
1	Mental health	752
2	Children and vaccines	496
3	COVID-19 booster vaccine	288
4	Pregnant	246
5	Foodborne	235
6	Research	226
7	Community health	223
8	Flu	216
9	Cancer	198
10	Monkeypox	197
11	Cardiovascular diseases	193
12	Public health	183
13	Climate	148
14	Sepsis	141
15	Masks	127
16	Vector	122
17	Health equity	108
18	Antibiotics	108
19	Ebola	92
20	Humanitarian	91
21	Smoking	85
22	RSV^a^	59
23	Sun damage	52
24	Noise damage	50

^a^RSV: respiratory syncytial virus.

### Engagement Analysis

Our dataset includes engagement metrics for each post, specifically the number of likes and user comments.

We calculated the average number of comments and likes for all posts in each topic. Multimedia Appendix 2 presents the average number of comments received per topic, while Multimedia Appendix 3 displays the average number of likes. Among the topics, those with the highest user engagement based on the average number of post comments were humanitarian issues, masks, and COVID-19. In terms of engagement measured by the average number of post likes, the leading topics were humanitarian issues, masks, and cancer. Multimedia Appendices 2 and 3 also show increased engagement for topics related to vaccines and COVID-19.

### Sentiment Analysis

We analyzed the sentiment in the text of the posts and comments and calculated the sentiment scores of the posts and comments, as described in the Methods section.

**Figure 2:**
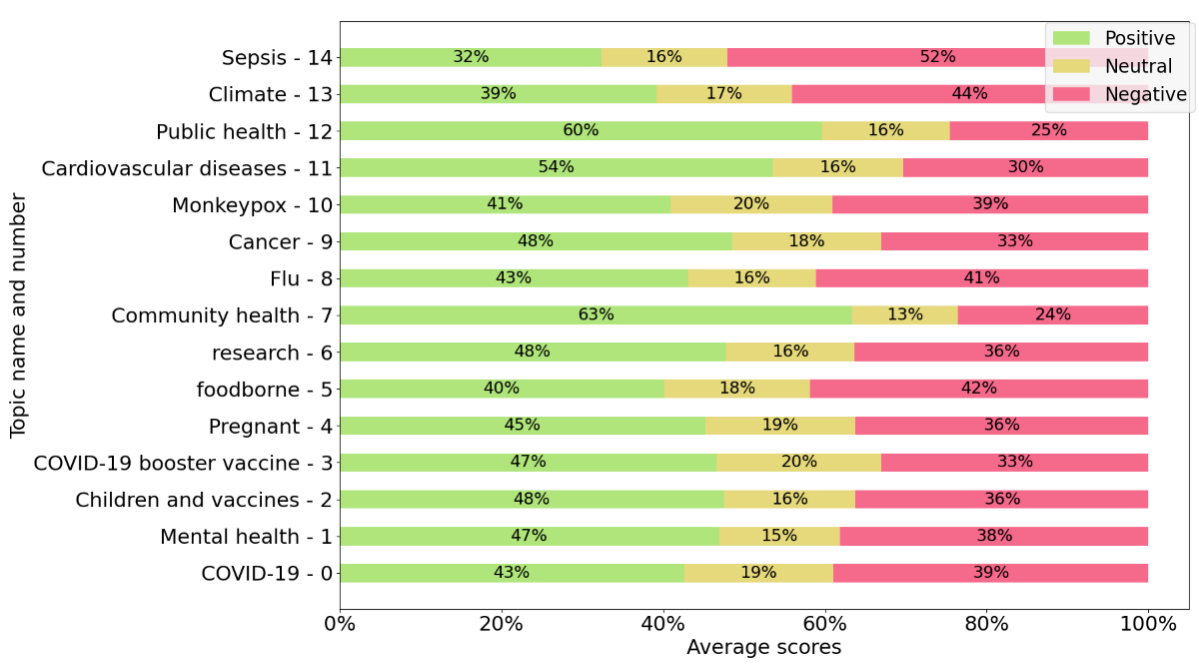
Sentiment scores of posts (average positive, neutral, and negative) for each topic.

**Figure 3:**
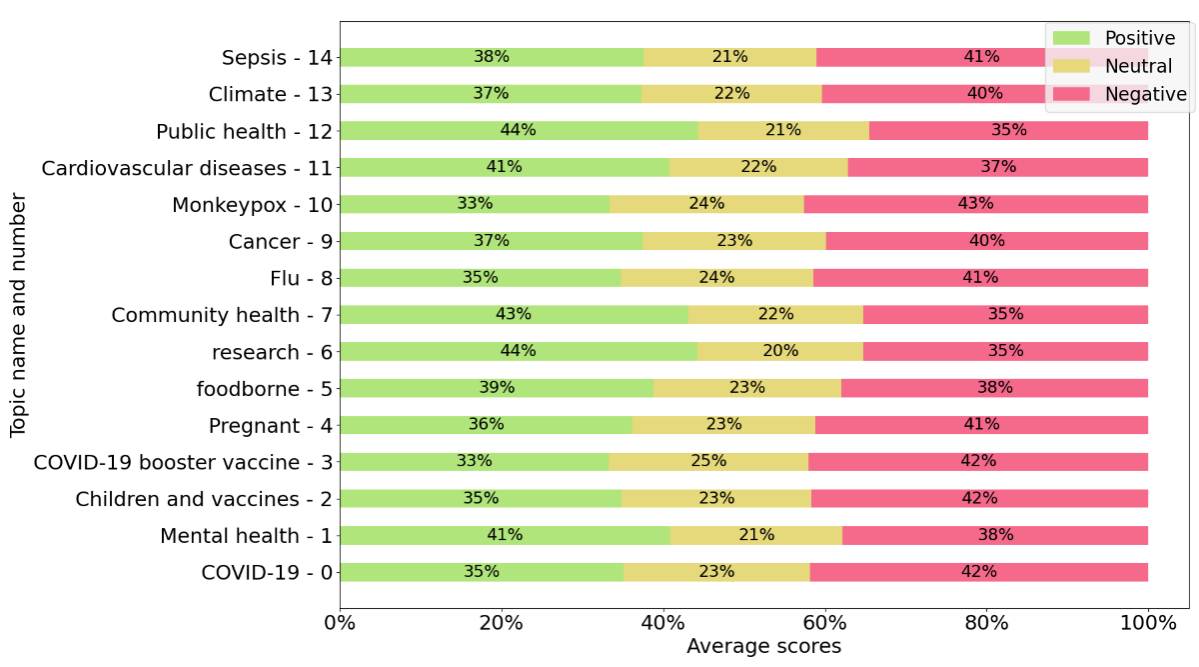
Sentiment scores of comments (average positive, neutral, and negative) for each topic.

Figures 2 and 3 present the average sentiment scores for health organizations’ posts and comments, respectively. In Figures 2 and 3, we selected only the 15 largest topics (those containing the greatest number of posts). Figure 2 shows that in certain topics, such as sepsis, climate, and foodborne illnesses, negative sentiment is the most prominent in the health organizations’ posts. However, in Figure 3, which presents the scores for the comments to the health organizations’ posts, we see that there are more topics where negative sentiment is dominant than in Figure 2, with the greatest negative sentiment found in the comments for posts about vaccines, specifically for posts in the COVID-19, children and vaccines, booster vaccines, and monkeypox topics.

While certain topics showed a dominant positive sentiment in the posts published by health organizations (as illustrated in Figure 2), this does not necessarily reflect how the public responds to those posts. The dominance of positive sentiment in these topics was based on the average sentiment scores, where the positive score was higher than both the negative and neutral scores. This indicated that these subjects were presented mostly positively by the organizations. However, when examining the sentiment expressed in user comments (Figure 3), we observed a contrasting response. In topics such as COVID-19, children and vaccines, and the COVID-19 booster vaccine, the comments exhibited predominantly negative sentiment, even though the organizations framed these topics positively. In these cases, the average negative score in the comments was higher than both the positive and neutral scores. In other words, while the organizations tried to communicate these topics in a positive light, the public’s reaction to them was largely negative.

### Emotion Analysis

We analyzed the emotion in the text of the posts and comments and calculated the emotion scores of the posts and comments, as described in the Methods section.

**Figure 4:**
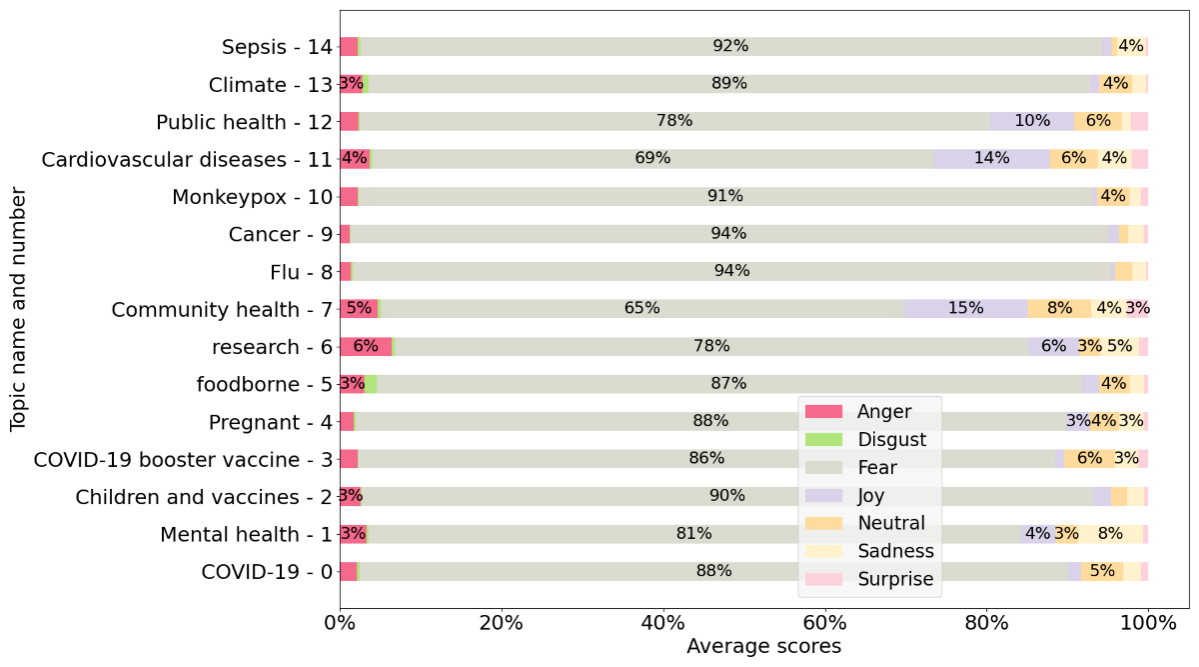
Emotion scores of posts (average anger, disgust, fear, joy, neutral, sadness, and surprise) for each topic.

**Figure 5:**
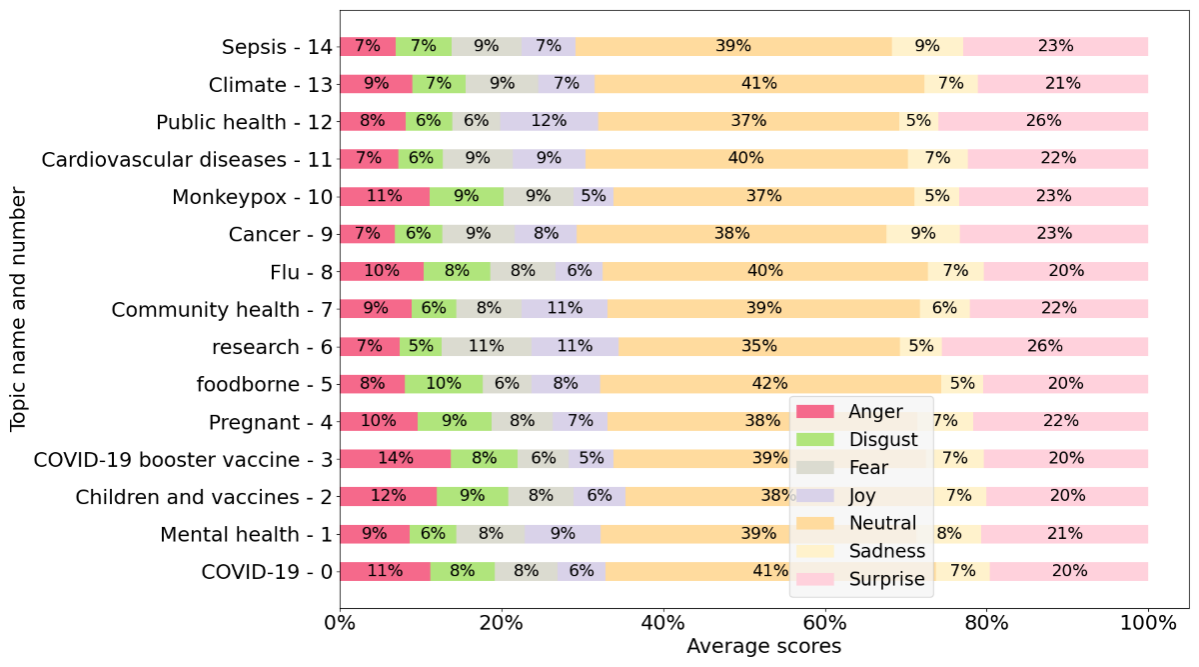
Emotion scores of comments (average anger, disgust, fear, joy, neutral, sadness, and surprise) for each topic.

The emotions present were anger, disgust, fear, joy, neutral, sadness, and surprise. Figure 4 presents the average emotion scores for the posts, and Figure 5 displays the average emotion scores for the comments. In Figures 4 and 5, we selected only the 15 largest topics (those containing the greatest number of posts).

As seen in Figure 4, the dominant emotion in all topics was fear, and its scores were higher than those of all the other emotions in the posts. In other words, the text in the health organization posts was characterized by a very high level of fear. Among the topics with the highest fear emotion were sepsis, monkeypox, cancer, and flu.

However, as shown in Figure 5, fear is not the dominant emotion; instead, neutral responses prevail. This suggests a disconnect between what health organizations consider fear-inducing issues and how the public actually responds, displaying less fear. When examining other emotions in Figure 5, we see that the topic generating the most anger is vaccines, with the COVID-19 booster vaccine specifically eliciting the highest levels of anger. In terms of sadness, cancer and sepsis were the topics that evoked the strongest feelings of sadness.

### Gap Analysis

Given the difference observed between the high average fear in the health organizations’ posts compared to the very low fear in the public’s responses to the posts, we examined the topics with the largest gap between the 2 average fears as revealed in the emotion analysis. For each topic, Table 4 presents the average fear in the posts of health organizations, the average fear in the comments, and the difference between the two. As can be seen in Table 4, the topics with the highest gap are masks and flu.

**Table 4:** Each topic’s average scores for fear in post and comments and the gap.

Topic name	Topic number	Fear in posts, mean (SD)	Fear in comments, mean (SD)	Gap
Masks	15	0.958 (0.072)	0.092 (0.166)	0.881
Flu	8	0.938 (0.156)	0.066 (0.180)	0.857
Vector	16	0.931 (0.090)	0.101 (0.196)	0.846
Cancer	9	0.936 (0.126)	0.062 (0.191)	0.846
Sepsis	14	0.917 (0.124)	0.106 (0.191)	0.831
Children and vaccines	2	0.904 (0.152)	0.086 (0.181)	0.823
Noise damage	24	0.913 (0.150)	0.084 (0.198)	0.821
Monkeypox	10	0.906 (0.138)	0.061 (0.197)	0.819
Ebola	19	0.898 (0.170)	0.085 (0.193)	0.812
Foodborne	5	0.870 (0.198)	0.077 (0.155)	0.810
Pregnant	4	0.879 (0.192)	0.086 (0.172)	0.803
Climate	13	0.893 (0.208)	0.090 (0.197)	0.803
COVID-19	0	0.876 (0.201)	0.060 (0.181)	0.799
COVID-19 booster vaccine	3	0.861 (0.178)	0.086 (0.155)	0.798
RSV^a^	22	0.897 (0.161)	0.087 (0.222)	0.796
Health equity	17	0.815 (0.223)	0.091 (0.147)	0.754
Antibiotics	18	0.835 (0.274)	0.081 (0.192)	0.751
Sun damage	23	0.800 (0.272)	0.081 (0.169)	0.734
Mental health	1	0.806 (0.283)	0.111 (0.188)	0.722
Public health	12	0.780 (0.281)	0.060 (0.152)	0.720
Smoking	21	0.777 (0.254)	0.076 (0.143)	0.715
Research	6	0.783 (0.218)	0.064 (0.240)	0.672
Humanitarian	20	0.724 (0.341)	0.081 (0.207)	0.618
Cardiovascular diseases	11	0.694 (0.332)	0.084 (0.192)	0.608
Community health	7	0.646 (0.331)	0.077 (0.178)	0.565

^a^RSV: respiratory syncytial virus.

### Linguistic Analysis

The linguistic analysis results are presented in Multimedia Appendix 4. It contains the top 50 phrases for unigrams, bigrams, trigrams, and four-grams in posts receiving predominantly positive or negative responses, along with their TF-IDF scores.

Results revealed that phrases associated with positive public responses promote public health awareness, vaccination benefits, and preventive measures. As an example, the phrases that were only included in the top 50 phrases of the health organizations’ posts that received positive responses included “stay healthy” “awareness month,” “signs symptoms,” “raise awareness,” “save lives,” “help slow spread,” “health care provider,” and “better health better understanding.” In contrast, phrases associated with negative sentiment related to vaccination efforts, outcomes, policy mandates, and health monitoring include terms such as “data tracker,” “vaccinated covid,” “dose vaccine,” “severe illness hospitalization,” “illness hospitalization death,” and “covid 19 vaccine booster.”

### Time Analysis

**Figure 6:**
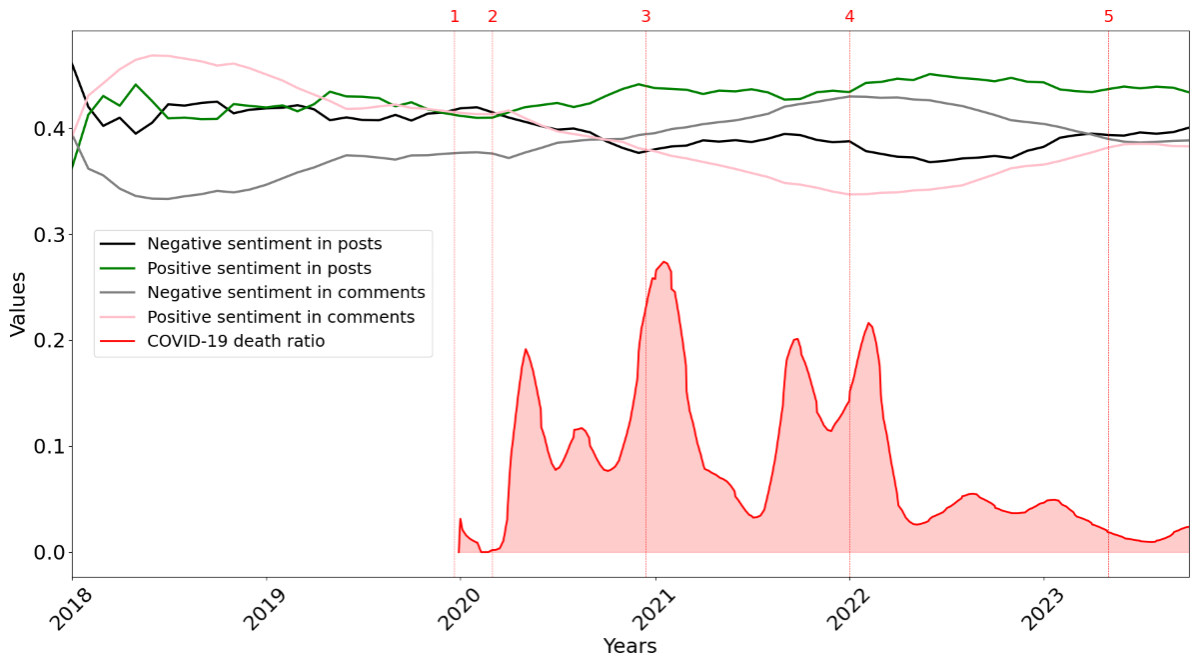
Evolution of sentiment (average positive and negative scores) in posts and comments over the years, combined with the COVID-19 death ratio.

**Figure 7:**
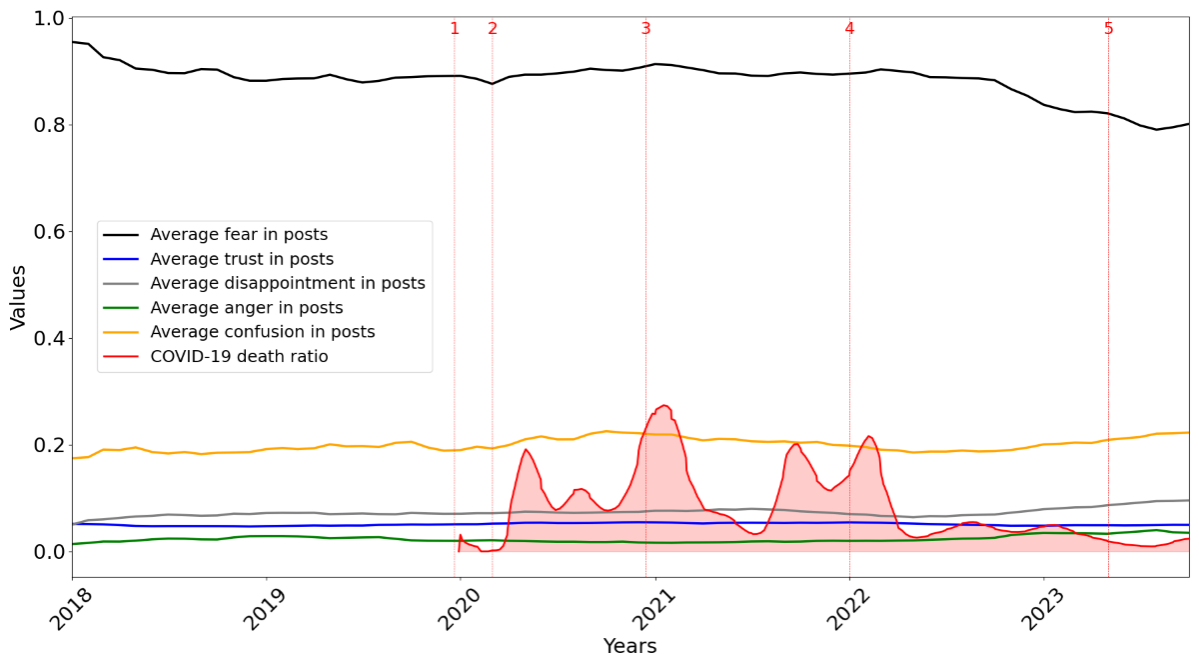
Evolution of emotion (average scores for trust, fear, anger, confusion, and disappointment) in posts over the years, combined with the COVID-19 death ratio.

**Figure 8:**
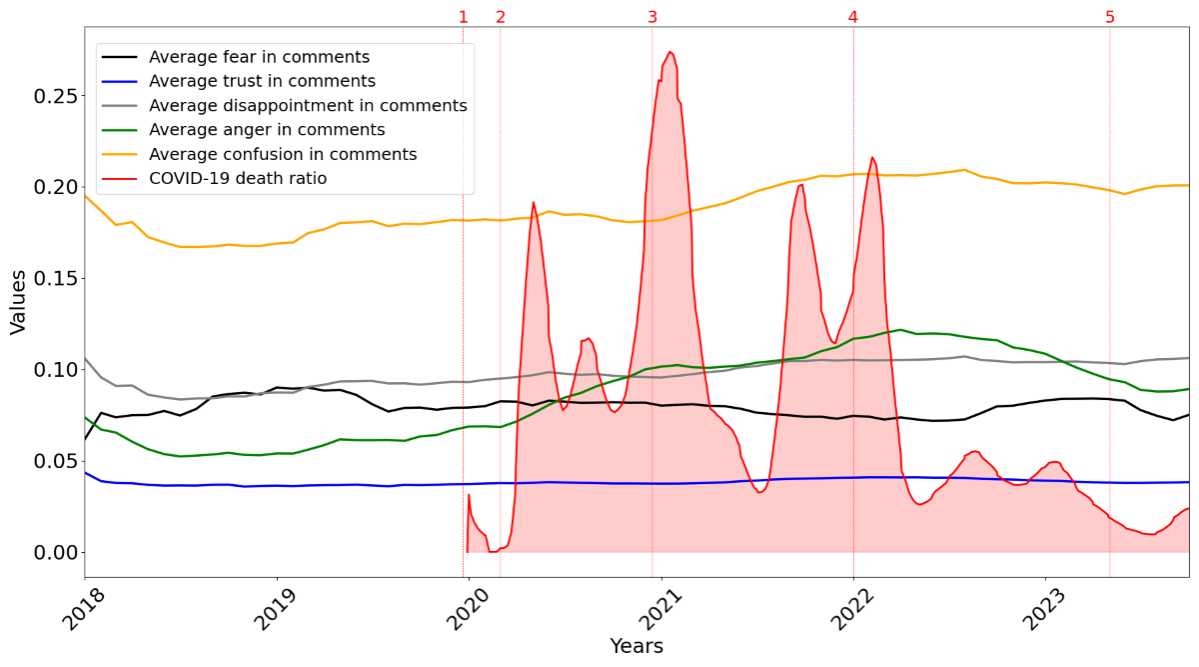
Evolution of emotion (average scores for trust, fear, anger, confusion, and disappointment) in comments over the years, combined with the COVID-19 death ratio.

Figures 6-8 illustrate sentiment and emotional shifts in posts and comments from 2018 to 2023 along with the COVID-19 death ratio. The numbers 1 to 5 in Figures 6-8 represent 5 significant milestones during the COVID-19 pandemic. These include the following: (1) first report of COVID-19 in late 2019, (2) the declaration of COVID-19 as a global pandemic by the WHO in March 2020, (3) the administration of the first COVID-19 vaccine in December 2020 in the United Kingdom, (4) the peak of the Omicron wave in early 2022, and (5) the WHO’s declaration in May 2023 that COVID-19 was no longer a global health emergency.

Figure 6 presents the average positive and negative sentiment expressed in posts and comments over time. The negative sentiment in comments increased over time and then began to decrease after the peak of the Omicron wave. Positive sentiment in comments showed a similar but opposite pattern, as it decreased and then increased after the peak of the Omicron wave. Regarding the sentiment of posts, it appeared that positivity in posts increased slightly over the years, while negativity decreased. The peaks of the COVID-19 death ratio aligned with increased negative sentiment in comments.

Figure 7 explores the average levels of emotions—fear, trust, disappointment, anger, and confusion—in posts. Fear consistently dominated posts and remained relatively steady until it declined, coinciding with a significant reduction in the death ratio. Trust, anger, and disappointment remained relatively steady throughout the years. Confusion gradually increased following the declaration of the pandemic.

Figure 8 shows the average levels of emotions—fear, trust, disappointment, anger, and confusion—in comments over the same period. Emotions of fear and trust remained relatively steady throughout the years. Anger and disappointment increased around death ratio peaks and, overall, showed a general upward pattern over the years. However, anger began to decline slightly after the Omicron peak and the subsequent decrease in the death ratio.

## Discussion

### Principal Findings

The findings of this study should be interpreted with consideration of the dataset’s geographic composition. As most of the health organizations analyzed are based in the United States, the topics emphasized may reflect US-specific health priorities, and the patterns of public engagement, sentiment, and emotional responses to these topics may also be shaped by US cultural and social contexts. The selected health organizations have a high number of followers on the platform, hold substantial influence, and reach audiences beyond national boundaries. Still, the framing and intensity of reactions observed in this study may not be fully generalizable to health organizations in other countries or regions, particularly because the characteristics of the users engaging with the content (such as their geographic location or demographics) are unknown.

Our findings on engagement analysis show that humanitarian issues, including the Ukraine war and the war in Gaza, and the COVID-19 pandemic received the greatest response from the public. This supports prior research, which has shown that those disasters, including humanitarian crises, such as nature-related crises and wars, are a major concern of the public and policy makers [101]. In recent years, conflicts, such as the ongoing Ukraine war and the war in Gaza, have had a significant impact on international stability, affecting economies, migration patterns, and security measures, particularly in Europe [102]. These conflicts have not only disrupted local societies but have also prompted reevaluation of humanitarian aid and intervention strategies globally. The connection between humanitarian conflicts and adverse health outcomes is well documented. Studies have shown increased incidence of mental health disorders, infectious disease outbreaks, and chronic health conditions in conflict-affected areas [103]. As a result, health organizations worldwide have recognized the urgent need to develop comprehensive strategies for emergency preparedness. This includes enhancing public health response capabilities, improving prehospital care, and integrating disaster medicine in public health frameworks to better address humanitarian crises [104,105].

The COVID-19 pandemic has fundamentally altered global perceptions and practices across various sectors, revealing both strengths and weaknesses in systems and policies worldwide [106]. During the COVID-19 pandemic, the public turned to health organization websites and social media channels to receive timely updates on infection rates, government guidelines, and evolving safety protocols [107,108]. Trust in organizations such as the WHO and CDC was crucial, as these bodies communicated essential information, adapting advice as new data emerged about the virus’s spread and impact. The results of this study further highlight that a significant portion of posts shared by health organizations focused on the topic of COVID-19. In addition, these posts generated substantial public engagement, demonstrating the public’s heightened interest and concern regarding COVID-19–related information. Given the profound impact of COVID-19, the public remains extremely interested in these insights, recognizing that understanding what was done well and what failed can better prepare societies for future health crises. Consequently, the ongoing dialogue among scientists, policy makers, and the public about these findings continues to shape postpandemic recovery strategies and fortify global health preparedness [109].

Our sentiment and emotion analysis of responses to health organizations’ posts showed that the highest percentage of negative scores was in topics related to vaccines and monkeypox. Moreover, our results show that the highest level of anger was observed in topics related to the COVID-19 booster and children’s vaccination in general. The high anger and negative sentiment can be attributed to vaccine hesitancy.

Vaccine hesitancy and antivaccine movements have long posed significant challenges to public health [110], particularly in communities with historically low trust in government and pharmaceutical institutions [111]. The analysis of topics associated with high anger emotions, such as COVID-19 vaccines, can be enriched by examining the emotional characteristics and motivations of specific user groups with vaccine hesitancy. For instance, women are generally more likely to exhibit vaccine hesitancy than men [112]. Alternatively, older individuals tend to display lower levels of hesitancy, likely due to their awareness of their vulnerability to COVID-19 complications, which may alleviate their fear or anger [112]. Vaccine hesitancy is also more prevalent among individuals with lower economic security [112]. Trust also shapes emotional and cognitive characteristics. Higher trust in health care providers, scientists, and global health organizations such as the WHO correlates with reduced vaccine hesitancy [112]. Conversely, in some contexts, higher trust in religious leaders is linked to increased hesitancy [112].

The COVID-19 pandemic has increased skepticism toward routine immunizations and increased hesitancy even among those previously compliant with vaccination schedules. The rapid development of COVID-19 vaccines, coupled with widely publicized adverse effects, heightened fears and deepened mistrust [113]. In addition, misinformation and conflicting narratives on social media further fueled fear, anger, and uncertainty, particularly in communities already skeptical of public health authorities. Our findings reflect these trends, showing increases in public negativity, anger, and disappointment over time.

Our linguistic analysis highlights that messages emphasizing safety, prevention, and public health benefits tend to elicit positive sentiments, while messages emphasizing vaccination efforts, outcomes, policy mandates, and health monitoring tend to elicit negative sentiments. This underscores the need for health organizations to refine communication strategies, focusing on clear, trust-building messaging to address concerns and counteract negative sentiments effectively.

Our analysis also reveals a gap between the fear conveyed in posts by health organizations and the public’s responses to these posts, particularly regarding the influenza vaccine and face masks during the COVID-19 pandemic. While health organizations intended to convey urgency, public comments did not reflect the same fear. Addressing skepticism and ambivalence about influenza vaccine and mask use is critical. Face masks are a critical tool in pandemic response [114], and enhancing public understanding of their effectiveness is essential for improving adherence and compliance during future outbreaks.

The difference in fear responses between the influenza vaccine and face masks can be understood by examining the interplay of social, psychological, and cultural factors [114]. One factor is psychological reactance, which occurs when individuals perceive mandates, such as mask requirements, as threats to their autonomy. This perception, combined with beliefs that masks are ineffective and an aversion to being forced to wear them, can trigger anger and counterarguments. Individuals with strong psychological reactance are particularly likely to exhibit these responses, reinforcing and intensifying their antimask attitudes [115,116]. Such resistance is further exacerbated by personality traits, which not only strengthen antimask sentiments but also link to broader vaccine skepticism, exaggeration of COVID-19 risks, and resistance to social distancing, often influenced by political conservatism [117].

Social and cultural dynamics also shape mask perceptions. For example, in many Western societies, masks are seen as extraordinary artifacts associated with emergency, while in Asian cultures, they are normalized as part of daily life [118]. In collectivist cultures, mask wearing aligns with a sense of duty to protect the community [116]. In addition, stigma, appearance concerns, and fears of being perceived as overly cautious further hinder acceptance [119].

Policy decisions and public health campaigns significantly influence perceptions. During the COVID-19 pandemic, inconsistent messaging from health authorities and varying guidance on mask use between countries and organizations contributed to public confusion and skepticism regarding their efficacy [120], despite substantial scientific evidence supporting their role in reducing transmission rates [120,121]. Therefore, consistent messaging and targeted communication strategies should focus on populations with antimask perceptions, aiming to reduce stigmas and address the underlying factors driving these attitudes.

Historically, the influenza vaccine has engendered significant hesitancy and objection from segments of the general public, a trend particularly evident among health care workers, who are critical in promoting vaccination [122]. Factors contributing to this hesitancy include misconceptions about vaccine efficacy, fears of adverse effects, and a perceived lack of urgency surrounding influenza, particularly when compared to more severe illnesses such as COVID-19 [123,124]. The emergence of the COVID-19 pandemic exacerbated this situation. During the COVID-19 pandemic, public focus shifted intensely toward COVID-19 vaccination campaigns, leading to a diversion of attention and resources from influenza vaccination efforts [125]. The flood of information regarding COVID-19 vaccines overshadowed the long-standing influenza vaccine campaigns. As a result, many individuals prioritized the COVID-19 vaccine over the seasonal influenza vaccine [125]. Data indicating that compliance with the influenza vaccine plummeted in several countries during 2023 and 2024 compared to prepandemic years highlight the ongoing challenges faced by public health authorities [125].

Despite the availability of the influenza vaccine, social media misinformation and evolving health narratives regarding influenza and COVID-19 have led to an atmosphere of uncertainty. For instance, some individuals may mistakenly believe that if COVID-19 variants pose health risks, the influenza virus may be less significant or that acquiring one vaccine negates the need for others. This shift necessitates a reassessment of public health strategies to reengage communities with the importance of influenza vaccination. Health organizations must develop targeted messaging that addresses misconceptions, enhances understanding of the influenza virus’s potential impact, and reinforces the protective benefits of vaccination, even during times when attention is focused on other diseases.

To summarize, in this study, we analyzed the quality of disseminated messages along 2 dimensions: public responses to topics and the emotions and sentiments expressed in those responses. Mapping these reactions to messages posted by health organizations is important for evaluating public engagement with specific health-related issues. Identifying emotional gaps can also help assess the effectiveness of health-related messages, revealing potential discrepancies between the importance health organizations assign to certain topics and the public’s perceived importance.

Our findings highlight gaps in fear responses regarding the influenza vaccine and wearing face masks during the COVID-19 pandemic, underscoring the need for transparent communication from health authorities and comprehensive education campaigns to address misconceptions and reassure the public. Understanding the psychological and social factors driving vaccine hesitancy after the pandemic is essential for tailoring effective public health strategies. Efforts must focus on fostering trust through consistent and clear messaging, transparency about vaccine development processes, and open forums for addressing public concerns. By doing so, it will be possible to mitigate misinformation, reduce fear, enhance public compliance, and improve vaccine uptake across populations.

### Limitations

Our study may have some limitations. We collected data from Instagram, and therefore, our results and conclusions are based on the posts and interactions on this social media platform. Our data did not include specific information about the users who commented on the health organizations’ posts. However, according to general information about Instagram users by Statista [126], Instagram had 2 billion monthly active users in 2024, with India leading the platform’s user base at approximately 360 million, followed by the United States with 169 million and Brazil with 134 million users. Younger users dominate the platform, with the 18- to 24-year age group being the largest demographic, followed by the 25- to 34-year age group. Participation declines significantly among older age groups, particularly those aged ≥55 years, who represent only a small fraction of the audience. Moreover, most users in the 18- to 34-year age group are men, while most users >34 years are women.

The absence of detailed user characteristics in our dataset may limit the generalizability of our findings, as biases could arise due to age, gender, educational level, or other demographic factors that influence engagement and sentiment patterns. Certain user groups may be overrepresented or underrepresented in the data, potentially shaping the interpretation of public responses. Future research should aim to systematically characterize respondents’ profiles, leveraging available metadata or incorporating external surveys to gain a clearer understanding of the audience engaging with health-related content.

The study also has limitations regarding the influence of social media algorithms on post visibility and engagement. The social media algorithms prioritize content based on user interactions, interests, and platform-specific ranking mechanisms, potentially introducing bias in public sentiment patterns. Nevertheless, the decision as to whether to engage and how to respond is left to the users. Therefore, although engagement data may not fully represent the broader public, it still offers insights into those actively participating in discussions. To mitigate bias, we ensured a diverse dataset by including a substantial number of posts from multiple health organizations.

It is important to acknowledge that the models used in this study may have limitations. Although BERTopic is an effective topic modeling technique, it assumes that each document (in our case, each post) is associated with a single dominant topic, which does not always reflect reality. As posts can discuss a variety of interconnected topics, it is often difficult to classify them accurately under a single theme. Furthermore, topic separation relies on clustering techniques, which may not always produce clear or optimal topic divisions, resulting in the merging of distinct topics or the fragmentation of related discussions, reducing interpretability.

Similarly, the sentiment and emotion models may inherit biases from their training datasets, influencing detection. These models are not always fully accurate and often struggle to capture context-dependent sentiment shifts, irony, and implicit emotional expressions, which may result in misinterpretations.

In addition, as previously mentioned, the health organizations in this study are predominantly US based, with limited representation from other countries. This concentration may influence the topics represented in the dataset, reflecting US-specific health priorities and cultural dynamics, as well as the public sentiment, emotional responses, and engagement observed in relation to those topics. Future research could incorporate data from a more geographically diverse set of health organizations, enabling cross-cultural comparisons and providing a broader understanding of global public health communication and audience responses.

### Conclusions

This study demonstrates the value of our methodology in assessing public responses and emotions expressed regarding health-related messages. By identifying emotional gaps, particularly fear, we were able to uncover discrepancies between the fear health organizations assign to issues and the fear that the public expresses in response. The greatest gaps were revealed with regard to influenza vaccines and face masks during COVID-19.

These findings emphasize the need for transparent communication and trust-building strategies that consider the emotional and sentiment-driven responses of the public on social media. By understanding the psychological and social dynamics of public interaction with health information, particularly regarding vaccine resistance, organizations can support public health goals, foster trust and efficient engagement, counter misinformation, and encourage informed health behaviors.

In future research, we plan to apply our topic modeling and sentiment and emotion analysis approach on other social media platforms to gain a more comprehensive view of the public’s response to the posts of health organizations across various digital platforms. In addition, we aim to extend our analysis beyond social media to offline settings, such as newspapers and television campaigns. Because public responses are essential to our method, we will also incorporate surveys to assess audience reactions and engagement with these campaigns. Investigating how fear-based messaging and public emotional responses manifest in both online and offline environments will provide deeper insights into the broader impact of health communication strategies. In addition, we plan to investigate misinformation within the comments on health organization posts and identify the topics where inaccuracies are prevalent.

## Appendix

Multimedia Appendix 1 The number, name, size, and top-5 most significant words of each topic.

Multimedia Appendix 2 Average number of post comments per topic. RSV: respiratory syncytial virus.

Multimedia Appendix 3 Average number of post likes per topic. RSV: respiratory syncytial virus.

Multimedia Appendix 4 Top-50 phrases for unigrams, bigrams, trigrams, and four-grams in posts receiving predominantly positive or negative responses with corresponding term frequency–inverse document frequency scores.

## Abbreviations

API: application programming interface
BERT: Bidirectional Encoder Representations from Transformers
CDC: Centers for Disease Control and Prevention
c-TF-IDF: class-based term frequency–inverse document frequency
MH: Ministry of Health
NHS: National Health Service
TF-IDF: term frequency–inverse document frequency
WHO: World Health Organization

## Data Availability

The datasets generated or analyzed during this study are available from the corresponding author on reasonable request.
