# Supplementary figures and images for: Exploring Topics, Emotions, and Sentiments in Health Organization Posts and Public Responses on Instagram: Content Analysis
Source: JMIR Infodemiology. 2025 May 2;5:e70576. doi: 10.2196/70576 (PMC12084776; doi:10.2196/70576)

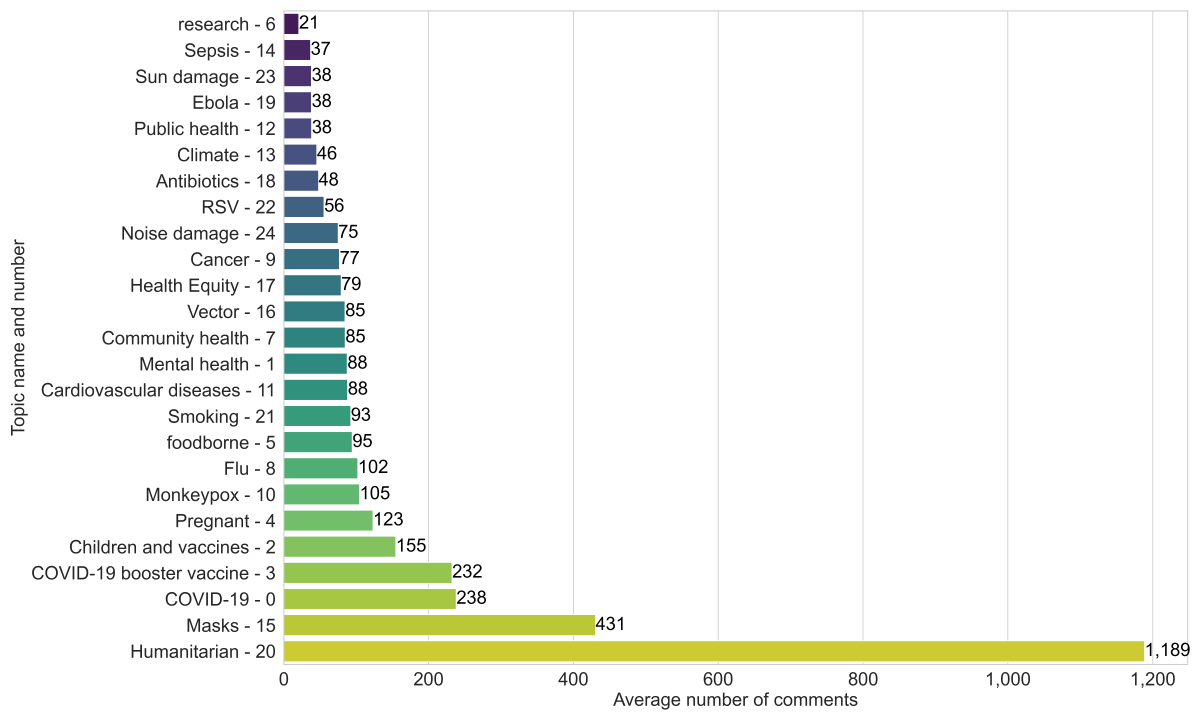

Supplement: Multimedia Appendix 2 [file infodemiology_v5i1e70576_app2.png]

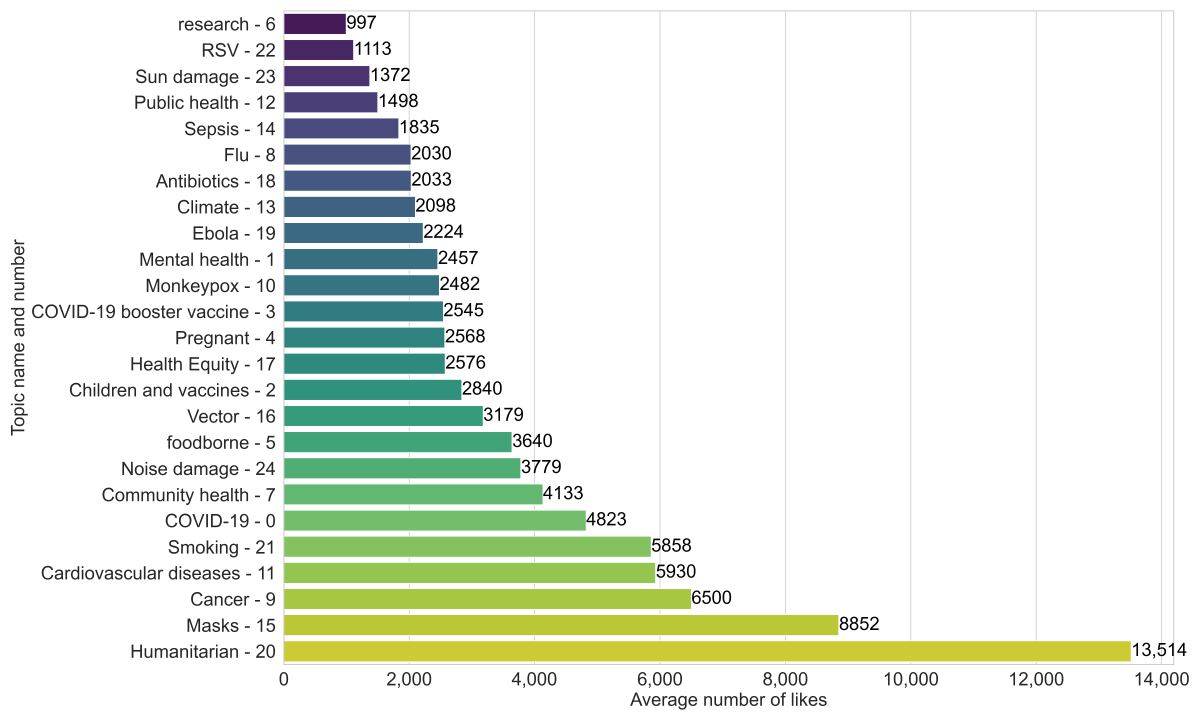

Supplement: Multimedia Appendix 3 [file infodemiology_v5i1e70576_app3.png]
